# Influence of Selenium and Mercury on Age-Related Cataracts in the Brazilian Amazon

**DOI:** 10.1289/ehp.1003242

**Published:** 2011-04

**Authors:** Claudio Minoia, Anna Ronchi, Paolo D. Pigatto, Gianpaolo Guzzi

**Affiliations:** Laboratory of Environmental and Toxicology Testing, “S. Maugeri” IRCCS, Pavia, Italy; Department of Technology for Health Dermatological Clinic, IRCCS Galeazzi Hospital, University of Milan, Milan, Italy; Italian Association for Metals and Biocompatibility Research–AIRMEB, Milan, Italy, E-mail: gianpaolo_guzzi@fastwebnet.it

In their article, Lemire et al. (2010) provided important data on the frequency of age- related cataracts among adults in the Brazilian Amazon and found a correlation between age-related cataracts and whole-blood total mercury (Hg) concentrations and selenium (Se) levels in plasma and whole blood. However, in the “Discussion” of their paper, they stated that they “observed no adverse effects although Se concentrations were very high, reaching 1,500 μg/L for [blood]-Se and 913 μg/L for [plasma]-Se” (Lemire et al. 2010). However, they did not mention the potential and substantial adverse health effects associated with a high body Se burden. There are potentially adverse consequences to Se body burden, such as hair loss (alopecia), tooth decay, nail changes, peripheral paresthesias, weakness, skin lesions, and diabetes (Hira et al. 2004; Nuttall 2006; Shearer 1975; Stranges et al. 2010; Sutter et al. 2008; Yang et al. 1983). It would be valuable to know what Se-related adverse effects were observed by Lemire et al. (2010). Most studies, taken together, suggest a possible attenuation of Hg toxicity, probably as insoluble form of Hg selenide (Clarkson 2002; International Programme on Chemical Safety 1990).

Selenium may be able to delay the onset of toxic effects in animal models exposed to methylmercury in the diet (Clarkson 2002; Ganther et al. 1972). However, the Hg–Se interaction may not have an equivalent effect in some animals. There is evidence that coadministration of methylmercury and Se may lead to an important synergistic effect (Heinz and Hoffman 1998). In studies of persons who have been co-exposed to Hg and Se, toxic effects of Se should be taken into account to discover the potential synergistic effect between Se and Hg.
